# GPT-Powered Chatbot-Based Positive Psychology Intervention for Well-Being Among Parents of Children With Autism Spectrum Disorder: Single-Arm Mixed Methods Study

**DOI:** 10.2196/85060

**Published:** 2026-03-09

**Authors:** Wen Zhang, Rachel Yim Fong Leung, Ka Ki Mak, Haoyan Ge, Tyrone Tai On Kwok, Ricky Van Yip Tso, Anni Wang, Haixia Ma, Janet YH Wong

**Affiliations:** 1 School of Nursing and Health Sciences Hong Kong Metropolitan University Hong Kong China (Hong Kong); 2 School of Education and Languages Hong Kong Metropolitan Univeristy Hong Kong China (Hong Kong); 3 Faculty of Education The University of Hong Kong Hong Kong China (Hong Kong); 4 School of Nursing Fudan University Shanghai China

**Keywords:** autistic children, autism, parental well-being, chatbot, positive psychology, digital intervention, ASD, ChatGPT, autism spectrum disorder

## Abstract

**Background:**

Parents of autistic children frequently experience elevated stress levels, depressive symptoms, and reduced well-being. Positive psychology interventions (PPIs) can strengthen resilience, and chatbots offer a scalable channel through which such skills can be delivered. However, evidence on the evaluation of large language model–guided PPI-based chatbots for this population is limited.

**Objective:**

This study evaluated the feasibility and acceptability of a GPT-powered chatbot (“Allie”). This study was designed to deliver culturally adapted PPIs to parents of autistic children and to explore their preliminary effects on well-being, depression, stress, and health-related quality of life.

**Methods:**

We conducted a single-arm mixed-methods pilot study with 19 parents with autistic children. These parents engaged with Allie for 2 weeks to complete 8 structured PPI exercises. The primary outcomes were feasibility (completion, ease of use, and practicality) and acceptability (multidimensional user ratings). Secondary outcomes were the World Health Organization–Five Well-Being Index (WHO-5), Patient Health Questionnaire-9, Perceived Stress Scale-10, and Short Form-12 Health Survey (version 2) Physical and Mental Component Summary scores. Outcomes were analyzed using paired *t* tests or Wilcoxon signed-rank tests. Optional postintervention interviews were analyzed using reflexive thematic analysis.

**Results:**

A total of 17 (89.5%) participants completed all the exercises, which indicated a high degree of procedural feasibility. There were also high ratings for ease of use and practicality (means 4.47/5, SD 0.70, and 4.32/5, SD 0.67, respectively). Acceptability was favorable (overall satisfaction mean=5.68/7, SD 0.70; prompt response time=6.37/7, SD 0.68). The WHO-5 score improved significantly from 32.84 to 46.11 (*t*_18_=2.48, *P*=.02; Cohen *d*=0.52). Changes in the Patient Health Questionnaire-9 (*z*=−0.49, *P*=.63; *r*=0.11), Perceived Stress Scale-10 (*t*_18_=−0.82, *P*=.43; Cohen *d*=0.12), and Short Form-12 Health Survey (version 2) Physical Component Summary (*t*_18_=−0.94, *P*=.36; Cohen *d*=0.18) and Mental Component Summary (*t*_18_=−0.89, *P*=.39; Cohen *d*=0.17) scores were not significant. Qualitative feedback (14/19) described benefits aligned with PPI mechanisms such as greater self-reflection, a more positive orientation, perspective-taking, emotional support, and coping skills. However, participants also suggested refinements, such as more natural conversation (colloquial Cantonese), shorter or less repetitive outputs, user-chosen sequencing with reminders and progress tracking, multimodal features, and autism spectrum disorder–specific resources.

**Conclusions:**

This pilot study revealed the feasibility, acceptability, and preliminary improvement in well-being (WHO-5) of a PPI-based GPT-powered chatbot, Allie, among parents of autistic children. Although there was no significant short-term change in other outcomes, the findings provide insights into design priorities, including personalization, conversational naturalness, multimodal content, and autism spectrum disorder–specific guidance. Larger, controlled trials with longer exposures and more diverse samples are needed to establish efficacy and durability.

**Trial Registration:**

ClinicalTrials.gov NCT06438120; https://clinicaltrials.gov/study/NCT06438120

## Introduction

### Background

Autism spectrum disorder (ASD) is a neurodevelopmental condition that is often diagnosed in early childhood and has become a growing public health concern worldwide. The World Health Organization estimates that approximately 1 in 100 children worldwide has ASD [1]. On the basis of official data from the US Centers for Disease Control and Prevention, approximately 1 in 44 children were diagnosed with ASD in 2018 [2]. In Hong Kong, the Census and Statistics Department’s General Household Survey for 2019 and 2020 revealed a prevalence rate of 1.4% for ASD among the local population younger than 15 years of age [3].

### Poor Mental Health Among Parents of Autistic Children

ASD is characterized by different levels of disability and challenges in social communication, cognitive growth, and emotional expression. Moreover, ASD includes limited, repetitive behaviors and interests, along with sensory processing problems [4]. Raising autistic children can be stressful and difficult for parents. This stress can increase the risk of mental health issues and other negative effects. Previous research has indicated that approximately half of parents of ASD children, including Chinese parents, report depressive symptoms [5,6]. Parents of autistic children also report lower well-being and quality of life related to health compared with parents of children without ASD [7].

Multiple factors affect the mental health of these parents. These factors include a perceived lack of control over their child’s behavior, worries about the child’s developmental progress and daily living skills, and the ongoing demands of caregiving. Time limitations, strained relationships with their children, and uncertainty about the child’s future add to parents’ stress [8,9]. These stressors foster modifiable psychosocial processes—strengthening perceived control and self-efficacy to counter helplessness [10], identifying and intentionally using personal and family strengths to navigate daily caregiving [11], applying positive reappraisal and meaning-making to cope with developmental uncertainties [12], and fostering hope and future-oriented goal setting to organize demands into attainable steps; and nurturing supportive relationships [13]. Orienting intervention content to these processes directly serves the objective of helping parents recognize and mobilize their strengths and manage stress more positively.

### Positive Psychology Interventions

To address these challenges, emerging evidence supports the use of positive psychology interventions (PPIs) [14,15]. While relatively few randomized controlled trials (RCTs) have tested PPI strategies among parents or caregivers of autistic children, different RCTs and systematic reviews have indicated that parent-focused interventions, including PPIs delivered digitally or in group formats, can enhance parental well-being and self-efficacy and reduce caregiver distress [16-19]. PPIs, proposed by Seligman et al [20], consist of evidence-based exercises aimed at fostering positive emotions, cognition, characteristics, and behaviors. PPIs focus on individuals’ strengths and resources, which distinguish them from traditional psychotherapies. PPIs are brief and require only a few sessions. PPI instructions are straightforward to follow, which allows for self-administration.

Research has shown that PPIs not only reduce depressive symptoms and stress and anxiety levels but also place equal emphasis on enhancing well-being among individuals with major depression [21]. A meta-analysis of 20 studies suggested that cognitive behavioral therapy is not superior to PPIs in reducing depressive symptoms but that PPIs significantly improve well-being [22]. The effectiveness of PPIs in improving well-being has been demonstrated in individuals with chronic pain and physical disabilities [23] and in undergraduate students [24]. Our research group applied PPIs among Chinese parents who lost their only child and reported that PPIs significantly improved their well-being [14]. Overall, we hypothesize that PPIs can improve well-being, reduce stress levels and depressive symptoms, and enhance the quality of life of Chinese parents of autistic children.

### Chatbot-Based Interventions to Promote Mental Health: Advantages

Digital health tools, especially chatbots, offer a scalable and accessible method for delivering the benefits of positive psychology skills programs to parents of autistic children. First, these tools are perceived as being accessible and offering a structured set of content that mimics real-life conversations with a supportive friend. This is especially important for these parents, who often face heightened isolation and reduced social support due to caregiving demands [5,7]. Second, access to effective psychological help can be limited by geographical barriers, a lack of resources, or social stigma [25]. Digital tools such as chatbots can help eliminate these barriers by providing easy access to proven psychological support. Chatbots are available 24/7 and can provide immediate feedback and support. Third, chatbots can technically collect data on users’ interactions, which can guide the improvement and adaptation of the interventions and thus can be tailored to meet personal needs.

Recent advances in large language models (LLMs) have expanded how chatbots can be used in different domains. In the context of parenting and autism, recent studies have begun to validate the utility of these models. For instance, Entenberg et al [26] demonstrated the feasibility of a menu-based chatbot microintervention for parents, achieving high levels of engagement and learning outcomes. More recently, the focus has shifted to generative artificial intelligence (AI)’s ability to handle complex queries. Almulla and Khasawneh [27] evaluated several LLMs, including GPT-4 and Google Gemini, and found them highly accurate and readable when these LLMs answered common questions from parents of autistic children. Similarly, McFayden et al [28] reported that ChatGPT (OpenAI) can provide accurate information to parents of individuals with ASD. Seo et al [29] highlighted the role of AI-driven chatbots in facilitating pediatric communication, further supporting the potential of this technology to bridge the gaps in child, parent, and provider interactions.

However, while these studies confirm that LLMs can effectively provide information and support communication, there remains limited evidence on their ability to deliver structured therapeutic interventions. Liu et al [30] recently identified key success factors for GPT-based PPIs, which suggested that retrieval-augmented generation can effectively enhance user engagement in general mental health contexts. However, peer-reviewed reports of LLM-guided chatbots delivering a fully structured PPI for parents of autistic children are scarce. Instead, prior ASD-related chatbots typically focus on information, diagnostic support, or education for autistic individuals [31-33]. To fill this gap, we developed one of the first chatbots (named “Allie”) to promote well-being in this population. The primary aim of this pilot study was to evaluate the feasibility and acceptability of Allie among parents. A secondary aim was to explore its potential impact on parents’ well-being, stress, depressive symptoms, and quality of life.

## Methods

### Study Design

This was a single-arm mixed-methods design to develop and evaluate a GPT-powered chatbot named “Allie.” The overall study period lasted from September 2023 to January 19, 2025. This study included an initial setup, participant recruitment, data collection, and qualitative interviews. We selected a single-arm design because this study was an early-stage feasibility and proof-of-concept evaluation that focused on acceptability and technical stability. This approach is consistent with the obesity-related behavioral intervention trials model for early-phase behavioral treatment development and suggests that user acceptability and technical stability be prioritized before we invest resources in an RCT [34]. This study was approved by the Research Ethics Committee of Hong Kong Metropolitan University (RD/2023/1.20).

### Development of Chatbot Intervention

Allie is a customized chatbot powered by OpenAI’s GPT-4o model, which was released in May 2024. This model was selected for its strong performance on Chinese and Cantonese tests [35]. This model has a 128k contextual window that supports multiturn therapeutic dialog without the loss of previous interactions. This linguistic capability allowed Allie to capture cultural nuances and idiomatic expressions from the parents of autistic children.

The intervention period was set at 2 weeks, during which time participants engaged in approximately 1 new exercise every 1 to 2 days. We used a 2-week schedule to minimize participant burden to test the feasibility and engagement with Allie. The objective of this pilot study was procedural feasibility rather than an estimation of maximal clinical effects.

To ensure therapeutic fidelity and reduce the risk of AI hallucination, we limited Allie’s outputs to evidence-based PPI content [20,21,36]. This content included 8 core exercises that were adapted and validated among Chinese parents [14]. The 8 core exercises were based on the Positive Emotions, Engagement, Relationships, Meaning, Accomplishment (PERMA) framework [37]; details of the PERMA framework and exercises are summarized in Multimedia Appendix 1. The structure of the exercises was built on foundational concepts such as identifying strengths before diving into exercises that require deeper reflection, such as gratitude or savoring the moment.

**Figure 1:**
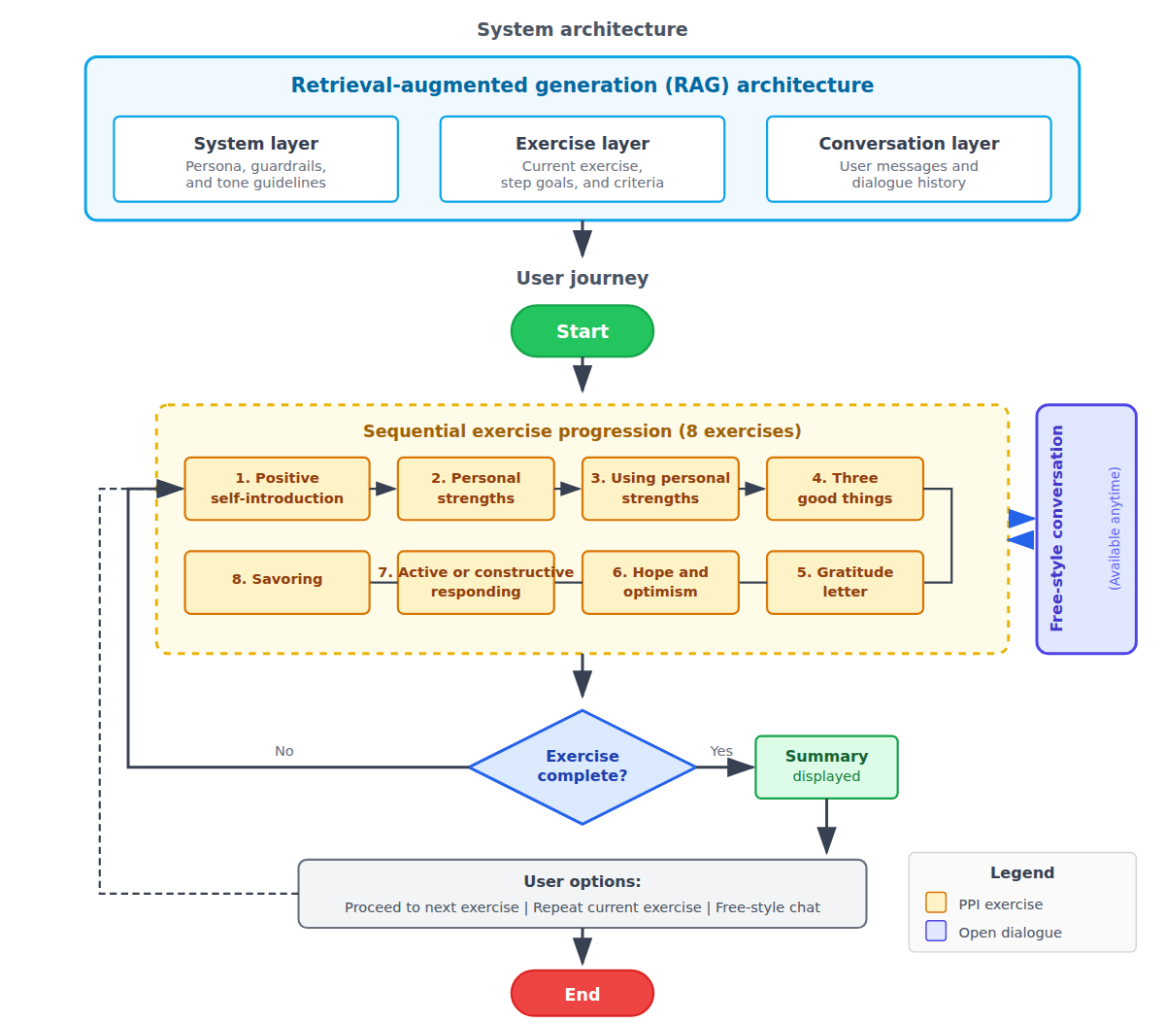
Allie system architecture and user flow. The system uses a 3-layer RAG architecture (system, exercise, and conversation layers) to deliver 8 sequential positive psychology exercises. After completing each exercise, participants received a summary and could proceed to the next exercise, repeat the current exercise, or engage in free-style conversation at any time. PPI: positive psychology intervention; RAG: retrieval-augmented generation.

To translate these 8 core exercises into interactive interventions that move beyond a simple prompt-based system, we implemented a retrieval-augmented generation architecture for Allie (Figure 1).

First, domain-knowledge grounding. The 8 core exercises were segmented into approximately 500-token passages and embedded with the text-embedding-3-large model. Passages and associated meta-tags (exercise ID, intended learning objective, and cultural notes) were stored in a vector database (Supabase pgvector). At runtime, each user’s turn triggered a semantic search (top-k=3); retrieved passages were prepended to the model’s context, grounding generation to validated content while allowing for conversational flexibility.

Second, prompt engineering workflow. We implemented the prompt stack in a 3-layer structure as follows:

1. System layer (approximately 300 tokens): this layer defines Allie’s persona (warm peer-mentor and uses written Chinese), guardrails (no diagnosis or medical advice, respect user autonomy, and refuse harmful requests), and tone guidelines drawn from the PERMA framework.
2. Exercise layer: this layer is a message block that encodes the current exercise, such as “using strengths,” step goals, and success criteria. When an exercise is completed, a system flag allows Allie to move forward automatically or wait for the user’s choice.
3. Conversation layer: in this layer, the full user message and the last 2 assistant responses are retained to maintain dialog coherence without exceeding the token limits.

**Figure 2:**
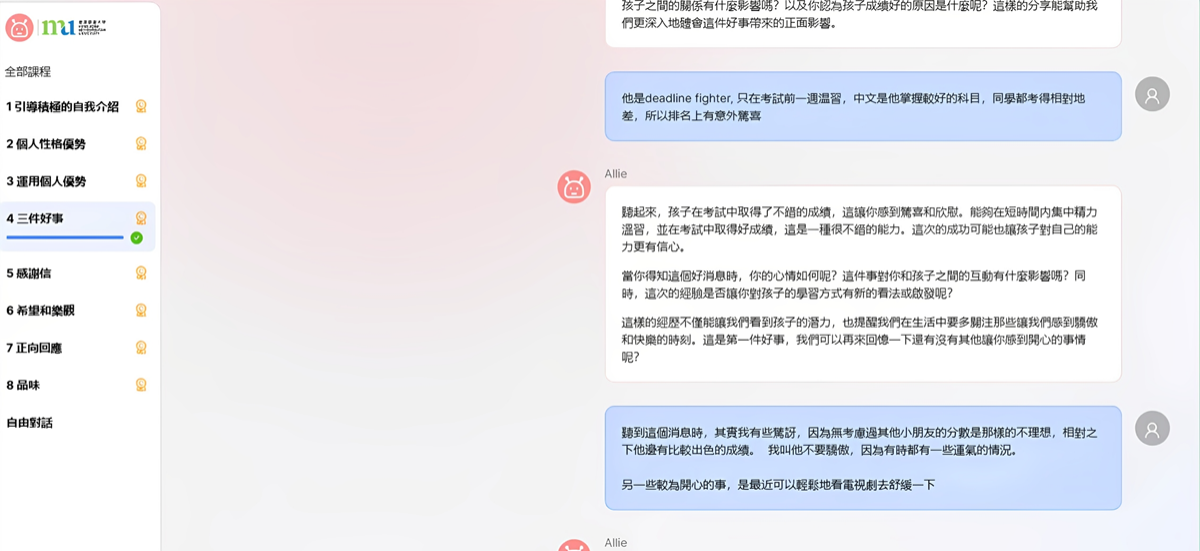
Parent-Allie chatbot conversation during the “three good things” exercise. This figure shows a standard interface. On the left sidebar, participants could select exercises 1 to 8 according to their progress or the “free-style conversation.” The corresponding content and dialog appear on the right. In this screenshot, the participant highlighted the following two positive experiences: (1) her son’s strong examination performance and (2) feeling relaxed after watching a television drama. Allie acknowledged these moments and prompted deeper reflection, asking how the child’s success and their interactions affected family dynamics and her mood. Allie encouraged the participant to value and cherish these proud and joyful moments. The full translation of the dialogue is documented in Multimedia Appendix 2. MU: Hong Kong Metropolitan University.

Through these steps, Allie learned to use a tone, phrasing, and content structure that reflected positive psychology principles such as promoting positive emotions and resilience. This approach allowed Allie to include cultural adaptations such as humility, which are important in Chinese culture. For instance, in the “three good things” exercise (Figure 2), Allie was programmed to encourage users to share positive moments in their lives.

Allie was designed to guide participants sequentially through the exercises. Participants could repeat or skip content on the basis of their preferences. Allie also extended its role from delivering structured content to “free-style conversation.” By using pretraining on a wide range of internet data, GPT-4o enabled Allie to generate coherent and empathetic responses to different user inputs. This ability allowed parent participants to discuss topics of their choice, such as real-life issues, emotional challenges, or their children’s homework, thus making use of the flexibility of LLMs.

### Data Security and Privacy

To safeguard data privacy, the research team ensured that no personal identifiers were sent to OpenAI. User IDs were created by partial digits of participants’ phone numbers; the transcripts were encrypted in transit (TLS 1.3; Transport Layer Security; Internet Engineering Task Force) and at rest (AES-256; Advanced Encryption Standard; US National Institute of Standards and Technology). This study obtained explicit consent for this data flow, which is consistent with the JMIR and CONSORT-AI (Consolidated Standards of Reporting Trials–Artificial Intelligence) transparency recommendations.

The pilot platform was access-controlled (password-protected) for privacy and security; therefore, the original URL is not publicly accessible. Representative screenshots are provided in Multimedia Appendix 3.

### Participants

Participants were parents who were providing long-term care for primary school-aged children (6-11 years of age) diagnosed with ASD. This age range was selected because the social and communication interactions of autistic children may be progressively more awkward during this period when social demands become more prominent [38]. Caregivers were eligible if they (1) self-identified as primary caregivers, (2) were aged 18 years or older, (3) were fluent in reading and communicating in Cantonese, and (4) had access to at least one mobile device with internet connectivity.

Caregivers were ineligible if their child did not meet the age range or ASD diagnostic criteria. Additionally, caregivers were excluded if they (1) had concurrent participation in a similar psychological intervention within one year, (2) were currently on regular psychotropic medications for mental health conditions, or (3) could not read or write in Chinese or Cantonese. No other exclusion criteria were applied.

As this was a feasibility study, no formal sample size calculation was needed. We estimated the sample size on the basis of the recommendation that a sample of 15 participants is appropriate for detecting a medium effect size. Assuming a 20% dropout rate, we planned to recruit 19 parents [39]. A sample size of 19 aligns with the recommendations for pilot trials [40,41]. These studies suggest that 12-20 participants are sufficient for estimating recruitment rates and variance for future power calculations.

### Procedure

A recruitment poster was distributed to NGOs, shared in Facebook (Meta) groups, and sent to parents who participated in our previous study. Interested parents contacted the research team and were screened against the eligibility criteria. Those who met all the inclusion criteria and provided written informed consent were enrolled. We then made arrangements for the baseline assessment. Upon enrollment, participants accessed Allie through standard web browsers [42] on their mobile phones or computers. While the intervention was designed to be self-paced, participants were required to complete the 8 core exercises sequentially within 14 days. We chose this brief schedule for 3 reasons. First, the primary aim of this pilot trial was to examine the feasibility and acceptability of the Allie chatbot rather than to estimate its maximal clinical efficacy. Methodological guidance on pilot and feasibility studies recommends using minimally burdensome protocols that are sufficient for testing recruitment, engagement, and study procedures [43,44]. Second, parents of autistic children often juggle intensive caregiving and employment responsibilities; thus, a shorter program was expected to be more acceptable and reduce attrition. Third, prior research has shown that brief PPIs, which last approximately 1 to 2 weeks, can improve well-being in other populations. Such interventions include 2-week “best possible self” writing; 2-week nostalgia, gratitude, or optimism exercises; and a 2-week nature-based noticing intervention [45-47]. Consistent with these precedents, we treated this 2-week exposure as a conservative starting point to test whether parents would engage with Allie. This approach also aimed to obtain initial estimates of attrition and user experience before a longer intervention was implemented.

### Measures

#### Overview

The following 2 categories of data were collected: demographic and outcome data. Demographic data were collected at baseline. Outcome data consisted of quantitative and qualitative measures. Assessments occurred at the following 3 time points: baseline (preintervention), immediately postintervention, and at an optional follow-up semistructured interview. Qualitative interviews were intended to gain deeper insights into intervention feasibility and acceptability, user experiences, and suggestions for future improvement.

#### Baseline Demographics

The demographic data included parent age, gender, education level, employment status, marital status, experience with AI, and household composition. Information about the age and gender of their autistic children was also included.

#### Primary Outcome Measures

##### Feasibility

We assessed feasibility by examining intervention completion rates, ease of use, practicality, and qualitative feedback. We used modified items from the Feasibility of Intervention Measure to measure ease of use and practicality [48]. These were 5-point Likert-type items with responses ranging from completely disagree (1) to completely agree (5). A high completion rate and user-reported ease of interaction with the chatbot were common indicators of feasibility in eHealth pilot studies [43,44]. Qualitative feedback on feasibility was obtained via semistructured interviews and is analyzed in the Results section.

##### Acceptability

Acceptability was measured with a custom questionnaire based on the work of Liu et al [49], 2023. Acceptability included several aspects of user satisfaction and perceived usefulness. These aspects included overall satisfaction, perceived appropriateness of session length and response time, adaptability and availability of the chatbot, relevance and trustworthiness of the information provided, interpersonal perceptions, privacy concerns, and intentions for future use. Items were rated on 5- or 7-point Likert scales depending on the dimension assessed.

#### Secondary Outcome Measures

##### Overview

Exploratory secondary outcomes were assessed using the following validated instruments.

##### Well-Being

The World Health Organization–Five Well-Being Index (WHO-5) measures subjective psychological well-being over the previous 2 weeks. This 5-item instrument uses a 6-point Likert scale (0 “at no time” to 5 “all of the time”), with raw scores converted to a 0-100 scale, where higher scores indicate better well-being. The WHO-5 is known for its brevity and strong psychometric validity [50]. Its Chinese version has also been validated [51].

##### Depression

The Patient Health Questionnaire-9 (PHQ-9) measures the severity of depressive symptoms. The PHQ-9 uses a 4-point Likert scale, where 0 indicates “not at all” and 3 denotes “nearly every day.” Total scores of 5, 10, 15, and 20 indicate mild, moderate, moderately severe, and severe levels of depression, respectively [52]. The reliability and validity of the Chinese version of the PHQ-9 have been validated [53].

##### Stress

The Perceived Stress Scale-10 (PSS-10) is used to assess perceived stress levels. The scale has 10 items that measure how individuals view situations in their lives as stressful over the past month. Each item is rated on a 5-point Likert scale ranging from 0 (“never”) to 4 (“very often”). Higher scores indicate greater levels of perceived stress. Its psychometric properties have been reviewed [54], and the Chinese version has been validated [55].

##### Quality of Life

The Short Form-12 Health Survey (version 2; SF-12v2) is used to assess health-related quality of life. This tool provides 2 summary scores—the Physical Component Summary score and the Mental Component Summary score. These scores reflect physical functioning, bodily pain, general health, emotional well-being, and role limitations due to physical or emotional challenges. The SF-12v2 includes 12 questions taken from the longer SF-36. The scores use norm-based T-scoring (mean 50, SD 10), with higher scores indicating better quality of life. The reliability and validity of the SF-12v2 have been proven in Chinese populations [56,57].

#### Qualitative Measures

Qualitative data were collected through optional semistructured interviews after participants completed the chatbot intervention. An interview guide was designed to explore users’ experiences with the intervention in an open-ended, conversational manner. Topics included users’ experiences and overall impressions, design and usability, perception of the exercises, emotional and behavioral impacts, and suggestions for future improvements.

### Data Analysis

#### Quantitative Analysis

Statistical analysis was performed using RStudio (versions 4.4.1 and 4.5.2; Posit Software, PBC). Descriptive statistics were used to summarize the demographics and baseline scores. Normality was checked using Q-Q plots and Shapiro-Wilk tests. Paired *t* tests were used for normally distributed outcomes; Wilcoxon signed-rank tests were applied for nonnormal distributions. We additionally reported change estimates with 95% CIs for each outcome—paired *t* test mean change for normal outcomes and Hodges-Lehmann estimate for the PHQ-9. To visualize individual-level change and heterogeneity, we also created paired dot plots for the WHO-5, which show each participant’s pre-post scores. Effect sizes were reported using Cohen *d* (paired *t* tests) or *r* (Wilcoxon tests) as appropriate.

#### Qualitative Analysis

Although the interview guide provided a general structure, participants were not prompted to respond in predefined conceptual domains. This analysis followed the 6-phase procedure outlined by Braun and Clarke [58] (2006). We used a bottom-up inductive approach to identify emerging patterns from participant responses without preconceived theories. The primary researcher manually coded the transcripts, and a second coder reviewed the initial codes in weekly peer-debriefing sessions to ensure reliability. Throughout this process, reflective memos were kept to document the evolving interpretation of the data. This approach followed the thematic analysis framework by Braun and Clarke [58], which helped identify themes on the basis of participants’ own expressions.

### Ethical Considerations

This study was approved by the Research Ethics Committee of Hong Kong Metropolitan University (Ref: HE-RD/2023/1.20) and conducted in accordance with the principles outlined in the Declaration of Helsinki. To protect privacy, no personal information, such as participants’ real names, addresses, or identification numbers, was collected. Each participant was assigned a pseudonymous code to replace identifiable data. However, participants’ phone numbers were retained solely to facilitate communication and follow-up during the study period. These phone numbers were stored securely and were not included in the analytical dataset. All participants signed written informed consent forms before enrollment. Participants received monetary compensation of up to HK $200 (US $25.6) for their participation in the study. Those who participated in the additional interview received an extra HK $100 (US $12.8).

## Results

### Participants

**Figure 3:**
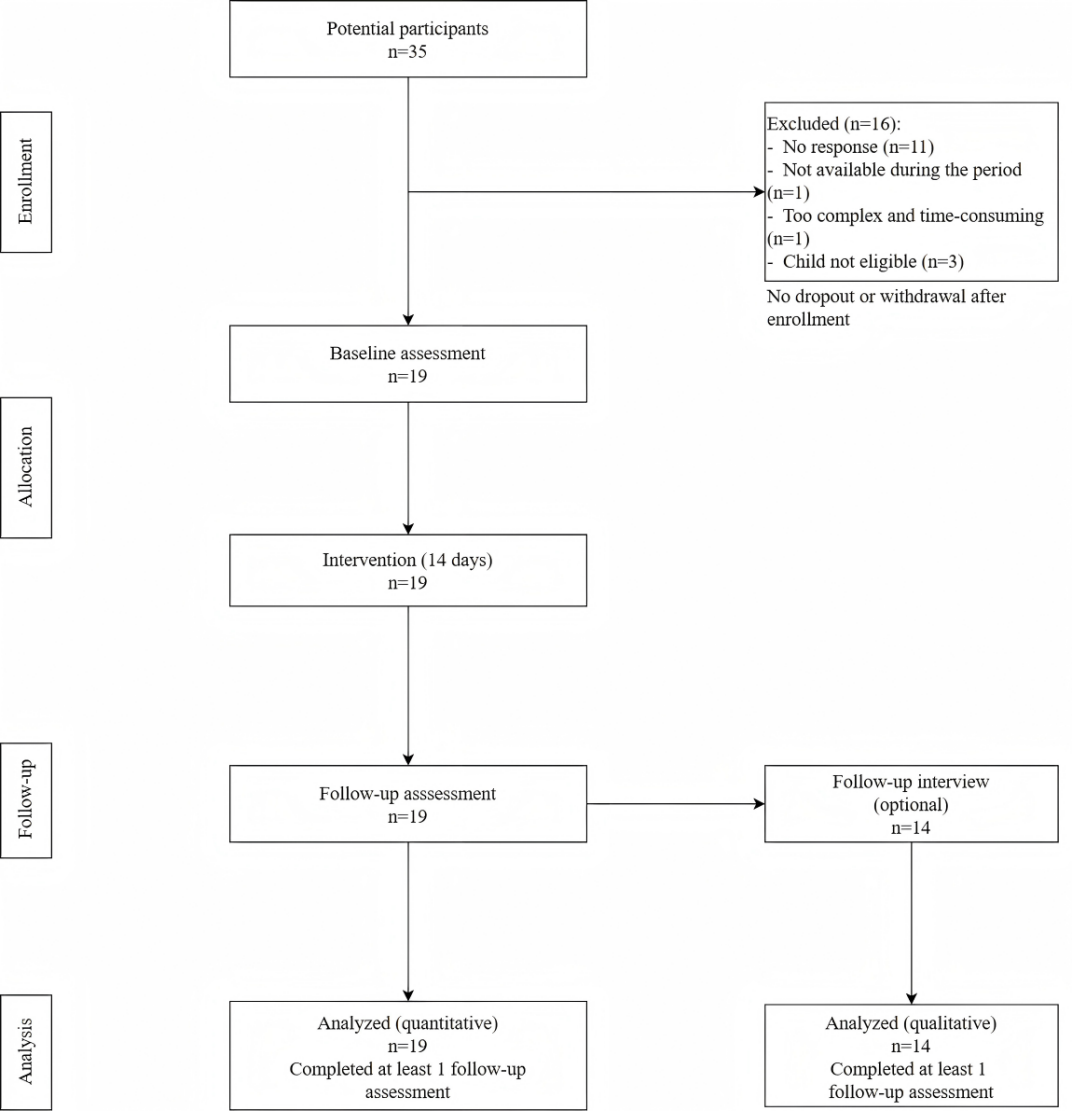
Participant flowchart.

A total of 35 parents were initially recruited for this study. Despite multiple outreach methods, recruitment for this population proved challenging due to practical barriers. A total of 3 were ineligible because their child did not meet the age or ASD diagnostic criteria. Among the remaining 32 eligible parents, 13 parents were excluded—11 did not respond to our follow-up contact, 1 reported being unavailable during the intervention period, and 1 declined to participate because the program was too complex and time-consuming for her. Nineteen eligible parents completed the baseline assessment and were enrolled in the 2-week intervention. No participants withdrew after enrollment (Figure 3).

The baseline characteristics of the participating parents are summarized in Table 1. All the participants were female (N=19, 100%). The parents’ mean age was 41.7 (SD 4.3; range 32-51) years. The majority had attained a bachelor’s degree (2/19, 36.8%), followed by high school education (5/19, 26.3%). With respect to experience with AI, 68.4% (13/19) of the participants had never tried AI before, while the remaining participants reported minimal to occasional AI use.

**Table 1:** Baseline characteristics of parent participants (N=19).

Characteristics		Values
**Parents**		
	Age (years), mean (SD)	41.7 (4.3)
**Gender, n (%)**		
	Female	19 (100)
**Education level, n (%)**		
	Middle school	1 (5.3)
	High school	5 (26.3)
	Diploma or associate’s degree	4 (21.1)
	Bachelor’s degree	7 (36.8)
	Master’s degree or above	2 (10.5)
**Employment status, n (%)**		
	Employee	5 (26.3)
	Self-employed	2 (10.5)
	Homemaker or unpaid family worker	12 (63.2)
**Marital status, n (%)**		
	Single or unmarried	2 (10.5)
	Married	16 (84.2)
	Separated	1 (5.3)
**Experience with AI, n (%)**		
	Never	13 (68.4)
	Occasional use	3 (15.8)
	Tried only a few times	3 (15.8)
**Caregiver support available, n (%)**		
	Yes	15 (78.9)
	No	4 (21.1)
**Children**		
	Age (years), mean (SD)	8.5 (1.4)
**Gender of children, n (%)**		
	Male children	17 (89.5)
	Female children	2 (10.5)
**Number of children in household, n (%)**		
	Only 1 child	10 (52.6)
	More than 1 child	9 (47.4)
**Number of autistic children in household, n (%)**		
	Only 1 child	17 (89.5)
	More than 1 child	2 (10.5)

Most participants (17/19, 89.5%) had 1 child diagnosed with autism at home. The mean age of the children was 8.5 (SD 1.4; range 6-11) years, and 89.5% (17/19) were male. Nearly 80% (15/19) of parents reported having additional caregiver support. With respect to employment, most parents were homemakers or unpaid family workers (12/19, 63.2%), while 5 (26.3%) were employed. Most participants were married (16/19, 84.2%).

### Quantitative Findings

#### Feasibility

We assessed feasibility along the following 3 dimensions: completion or attrition, practicality, and ease of use. The qualitative feedback related to feasibility is reported in the Qualitative Findings subsection.

#### Completion and Attrition Rate

The completion rate for the chatbot intervention exercises was high among parent participants. A total of 17 of 19 (89.5%) parents completed all 8 exercises within 14 days. Further, 2 (10.5%) participants completed only half of the exercises. The main reason reported was “forgetting to complete” (Table 2).

**Table 2:** Completion rate and reasons for incomplete participation (N=19).

Completion status		Values
**Rate of completion, n (%)**		
	Completed all exercises	17 (89.5)
	Completed half of the exercises	2 (10.5)
	Less than half of the exercises	0 (0)
**Reasons for incompletion, n (%)**		
	Forgot to complete	2 (100)
	No time	0 (0)

#### Practicality and Ease of Use

Parent participants found the chatbot easy to learn, as indicated by a mean ease-of-use score of 4.47 (SD 0.70) on a 5-point scale. They also highly rated its practicality, with a mean score of 4.32 (SD 0.67) for applicability to real-world settings.

#### Acceptability

Acceptability was evaluated by 7 dimensions that are composite scores. These scores are presented in Multimedia Appendix 4.

Overall, participants (N=19) reported that the chatbot intervention was acceptable. The mean level of satisfaction was 5.68 (SD 0.70) on a 7-point scale. A total of 18 (94.7%) participants reported that the duration of the exercise was appropriate. The mean perceived response promptness was 6.37 (SD 0.68). Participants rated the information provided as relevant (mean 5.82, SD 0.80) or trustworthy (assurance, mean 5.80, SD 0.70). They showed strong intentions to continue using the chatbot (usage intention, mean 5.68, SD 0.95). Adaptability (mean 5.53, SD 1.07) and empathy (mean 5.32, SD 1.15) received slightly lower ratings. Privacy concerns were also low (mean 4.43, SD 1.43), with lower scores indicating fewer concerns.

#### Exploratory Outcomes

Table 3 presents the pre-post outcomes and change estimates with 95% CIs. The mean increase in the WHO-5 score was 13.26 points (SD 23.31, 95% CI 2.04 to 24.49; t_18_=2.48; *P*=.02; Cohen *d*=0.52). Individual changes were heterogeneous, as reflected in the paired dot plot (Multimedia Appendix 5), ranging from –24 to +72 points. Most participants experienced small changes, while a smaller subset showed larger improvements.

**Table 3:** Pre- and postintervention scores for parent participants (N=19).

Measure	Pretest mean (SD)	Posttest mean (SD)	Pretest median (IQR)	Posttest median (IQR)	Test statistic	Change estimate (95% CI)^a,b^	*P* value	Effect size
PHQ-9^c^	N/A^d^	N/A	10 (14)	6 (7)	–0.49^e^	–0.50 (95% CI –2.50 to 1.50)	.63	0.11^f^
PSS-10^g^	20.53 (6.74)	19.74 (6.89)	N/A	N/A	–0.82 (18)^h^	–0.79 (95% CI –2.82 to 1.24)	.43	0.12^i^
WHO-5^j^	32.84 (24.86)	46.11 (26.00)	N/A	N/A	2.48 (18)^h^	13.26 (95% CI 2.04 to 24.49)	.02^k^	0.52^i^
SF-12^l^ PCS^m^	45.04 (8.40)	43.36 (9.91)	N/A	N/A	–0.94 (18)^h^	–1.69 (95% CI –5.45 to 2.07)	.36	0.18^i^
SF-12 MCS^n^	42.68 (8.65)	40.81 (11.88)	N/A	N/A	–0.89 (18)^h^	–1.86 (95% CI –6.27 to 2.54)	.39	0.17^i^

^a^Change estimate (95% CI) refers to the paired *t* test mean change for normal outcomes; the Hodges-Lehmann estimate for the Patient Health Questionnaire-9.

^b^For the Patient Health Questionnaire-9 and Perceived Stress Scale-10, a negative change indicates symptom reduction (improvement); for the World Health Organization–Five Well-Being Index and Short Form-12 Health Survey, a positive change indicates improvement.

^c^A Wilcoxon test was used because of the nonnormal distribution posttest (Shapiro-Wilk *P*=.04; see Multimedia Appendix 6).

^d^N/A: not applicable.

^e^z: standardized test statistic.

^f^Effect size *r*.

^g^PSS-10: Perceived Stress Scale-10.

^h^*t* test (degrees of freedom).

^i^Effect size *d*.

^j^WHO-5: World Health Organization–Five Well-Being Index.

^k^*P*<.05.

^l^SF-12: Short Form-12 Health Survey (version 2).

^m^PCS: Physical Component Summary.

^n^MCS: Mental Component Summary.

Changes in depressive symptoms, perceived stress levels, and physical and mental health–related quality-of-life scores were not significant. Specifically, depressive symptoms measured by the PHQ-9 decreased slightly (Hodges-Lehmann estimate –0.50, 95% CI –2.50 to 1.50; Wilcoxon z=–0.49; *P*=.63; *r*=0.11). Similarly, perceived stress (PSS-10) changed minimally (mean –0.79, SD 4.20, 95% CI –2.82 to 1.24; t_18_=–0.82, *P*=.43; Cohen *d*=0.12). With respect to health-related quality of life, the SF-12v2 physical and mental component scores decreased slightly after the intervention, but the changes were not significant (Physical Component Summary mean –1.69, SD 7.84, 95% CI –5.45 to 2.07; t_18_=–0.94; *P*=.36; Cohen *d*=0.18; Mental Component Summary mean –1.86, SD 9.11, 95% CI –6.27 to 2.54; t_18_=–0.89; *P*=.39; Cohen *d*=0.17). See Multimedia Appendix 6 for statistical assumption checks.

### Qualitative Findings

#### Perceived Feasibility

Participants’ responses echoed the quantitative feasibility results. Among the 19 participants, 14 joined the optional interview and completed the 8 core exercises. They commonly highlighted the chatbot’s user-friendliness and practicality. For instance, as 1 mother noted, “I think it’s quite good because it really feels like I’m chatting with a real person” (Participant 5, a homemaker aged 44 years who has no experience using AI). These verbatim excerpts illustrate high perceived usability and engagement. The key characteristics of the participants are listed in Multimedia Appendix 7.

#### Most Helpful Exercises

**Figure 4:**
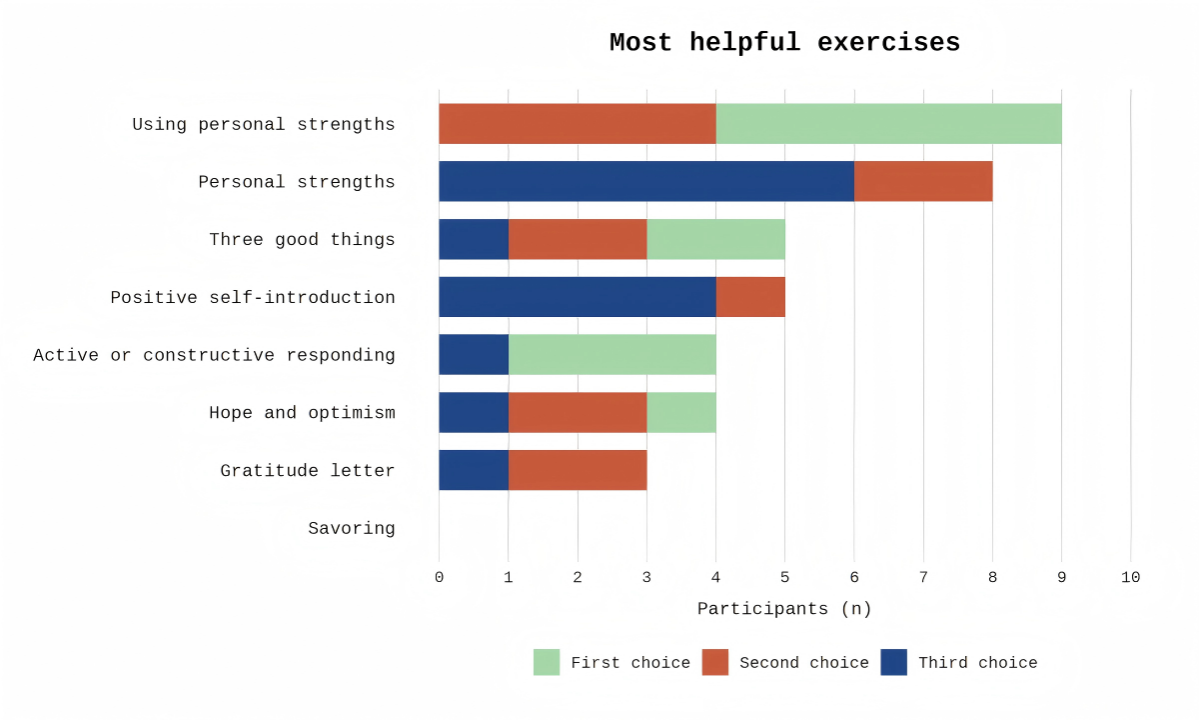
Most helpful exercises identified by participants.

When asked to identify the most helpful components, participants prioritized exercises focused on strength-building. “Using personal strengths” and “personal strengths” were the most frequently cited favorites among the 8 exercises (Figure 4). The table showing the choices of participants is in Multimedia Appendix 8. These exercises helped them rediscover capabilities that had been overshadowed by their caregiving demands.

#### Thematic Analysis of User Experience

Thematic analysis of the 14 semistructured interviews revealed participants’ perspectives on the chatbot intervention, which were categorized into the following 6 major themes.

### Theme 1: Fostering Self-Reflection and Insight

Many parents described how the exercises prompted them to pause and reflect more deeply on their daily lives. For instance, participant 13 (an employed mother aged 51 years) reflected on the “gratitude letter” exercise as follows:

It actually flips things around and makes you think about why you want to thank this person... It is a really good opportunity to reflect on this person or this event and why it matters so much to you.

Similarly, participant 5 (a homemaker aged 44 years with no AI experience) explained that Allie’s guided prompts encouraged her to notice aspects of her daily life she might otherwise overlook. She realized how her usual communication style might be perceived as “negative” or a “conversation stopper.” This suggests that Allie functioned as an “interactive mirror,” making implicit habits explicit, and that Allie supported reflective work by lowering the emotional barrier through stepwise questioning rather than requiring parents to generate insight unaided. Shorter excerpts are provided here, with more verbatim quotes in Multimedia Appendix 9.

### Theme 2: Promoting Positive Psychology Orientation

Participants consistently noted a shift from deficits and stressors toward positive moments. After completing an exercise, participant 3 (an employed mother aged 40 years with no AI experience) said the following:

Oh yes, there are things I should be grateful for. It really changed some of my thinking.

Beyond “thinking positive,” some parents described a more compassionate reappraisal of negative experiences; an example is as follows:

Even when there were unhappy or negative thoughts, it helped reframe them with more understanding and empathy.

Participant 6, a homemaker aged 35 years with minimal AI experience

Taken together, these accounts suggest that the chatbot did not go beyond eliciting positive statements and helped caregivers reframe their thoughts and emotions when they were dealing with ongoing stress.

### Theme 3: Offering New Perspectives

Several parents reported that Allie’s questions and feedback helped them reframe challenges and that it broadened problem-solving options and reduced cognitive narrowing. Parent 1 (a self-employed mother aged 42 years) described moments of realization as follows:

It would say, ‘Actually, you could try doing this,’ and I realize, ‘Yes, that’s right. Why didn’t I think of that myself?’

Another participant echoed this view. She highlighted that new perspectives came from challenging blind spots rather than replacing the parents’ own thinking, as follows:

Everyone has blind spots… it helps me move beyond my narrow thinking… it might give me a new direction.

Participant 11, a homemaker aged 47 years who occasionally used AI

This theme shows that the chatbot offered more than emotional reassurance; it helped parents adopt new perspectives on which they could act.

### Theme 4: Providing Emotional Support

A recurring experience involved feeling heard, comforted, and supported. Participant 2 (a homemaker aged 43 years with no AI experience) explained this as follows:

It felt like someone was comforting me... When I thought about its presence, I would chat with it a little, at least to help pull myself back up and not let myself sink too deeply. The more I thought about it, the heavier it felt, and then, I would start to feel like crying.

Another participant described mood repair through validation and positive reframing as follows: “...realizing you’re not as bad as you thought. Your mood got better. Then, it gave me lots of positive ideas, and that helped improve my mood” (Participant 8, a homemaker aged 32 years with no AI experience). She also noted Allie’s role as an accessible outlet when human support was unavailable as follows:

At times when I was overwhelmed and unsure how to cope and when I had emotions I wanted to express but it isn’t convenient to share them with others, I would turn to it.

These accounts indicate that caregivers perceived emotional support through the chatbot’s immediacy, responsiveness, and nonjudgmental tone, particularly when time and access to supportive conversations were limited.

### Theme 5: Developing Coping Skills

Parents also reported acquiring or strengthening coping strategies through exercise. Participant 1 noted that the program helped her manage her temper with her child as follows:

I felt less angry... I tried to hold it in as much as possible. After getting through that moment, the situation would usually feel better.

Similarly, participant 14 (a homemaker aged 42 years with no AI experience) agreed that stress-management techniques were practical for managing emotional challenges.

These coping approaches could be applied to both parents and their children. Participant 4 (a homemaker aged 46 years with no AI experience) emphasized that practical coping strategies mattered on 2 levels as follows:

How to help my child, how to… help myself too; these are personally very important.

These accounts indicate that the chatbot supported skill generalization—parents were not only reassured at the time but also gained the language and steps they could reuse in future stressful situations. Other verbatim quotes are presented in Multimedia Appendix 9.

### Theme 6: Recommendations for Future Improvements

Despite positive feedback, participants also recommended enhancements for future iterations of Allie. First, several participants wanted more human-like interactions. For example, participant 4 felt that colloquial Cantonese would be preferable, stating the following:

I think a more spoken Cantonese-style tone would be better.

Conversational continuity was another key expectation. Participants wanted Allie to acknowledge what was just said rather than repeating or asking questions in a generic way, as follows:

If it were a real person, they would acknowledge what you had just said. (Participant 13)

Smaller content segments instead of long messages were also suggested for human-like interactions. As participant 5 commented,

I mean, it shouldn’t send a whole long block all at once. It could split the content into parts and send it out in two or three messages.

All these human-like requests were intended to make Allie more natural, less burdensome, and more practically supportive in daily caregiving routines.

Another recommendation was greater flexibility and personalization in the program structure. Some parents proposed allowing parents to choose exercises on the basis of their needs rather than following a fixed sequence:

So, I think it would be better if it were more flexible, letting you choose what you want to do (Participant 6).

One parent also suggested tailoring recommendations on the basis of their interaction with Allie. This implies that perceived usefulness can increase when the chatbot helps users select the “right” exercise at the “right” time.

Additionally, participants proposed engagement-enhancing features (eg, role-play practice, visuals, optional audio, and emojis) and practical reminders. As 1 participant noted, “I would be afraid of forgetting” (participant 3). These requests suggest that usability improvements were not only aesthetic but also related to adherence and practicality in the real world.

Several parents also requested more concrete external resources, such as parenting guides, suggestions for suitable family activities, and access to community support services. These resources were seen as a way to supplement psychological support and better meet parents’ practical needs in real-world settings.

## Discussion

### Principal Findings

This pilot study evaluates one of the first generative LLM-based PPIs for parents of autistic children. The Allie chatbot demonstrated high degrees of feasibility and acceptability, which is evident in the 89.5% (17/19) completion rate across 8 exercises and favorable ratings for practicality and ease of use. Participants also reported significant improvements in their well-being, as measured by the WHO-5. Given the single-arm design and small sample size, this improvement should be interpreted as preliminary and hypothesis-generating. Additionally, there were no significant changes in depressive symptoms (PHQ-9), stress levels (PSS-10), or health-related quality of life (SF-12v2) during the 2-week intervention.

The qualitative findings provided deeper insight into the user experience. These findings indicated that Allie functioned as a supportive companion and facilitated core PPI processes. Parents described benefits that map closely to established PPI mechanisms, such as fostering greater self-reflection, promoting a more positive orientation, encouraging perspective-taking, providing perceived emotional support, and practicing new coping skills. Despite the positive feedback, participants also offered clear recommendations for refinement. They expressed a clear preference for a more personalized, interactive, and resource-rich virtual assistant rather than for a simple scripted chatbot.

### Comparison With Prior Work

This study differs from previous related research in 2 areas. First, some digital interventions for parents of autistic children have incorporated rule-based menu-style conversational components (eg, a virtual counselor delivered via menu-list options) [59]. While these systems can be helpful, they are often limited to setting conversation paths and do not have the flexibility to manage various user inputs. Second, earlier studies using LLMs in the ASD field have concentrated on applications for professionals, such as creating care plans or diagnostic comparisons [33,60,61], instead of offering a parent-facing and interactive therapeutic intervention. By embedding culturally adapted Cantonese PPI scripts in GPT-4o, Allie offers a structured, dynamic, and novel experience. This approach highlights a scalable, culturally aware option for the mental health care of parents with autistic children.

Our findings align with and extend recent research on AI-driven interventions. Consistent with the findings of Entenberg et al [62], who reported high levels of engagement (66.3% completion) with a menu-based parenting chatbot, our study revealed an even higher completion rate (89.5%) with the use of a generative interface. This finding suggests that the conversational flexibility of LLMs may further lower barriers to engagement even for highly stressed groups such as caregivers. This is particularly important for busy parents to overcome common barriers such as time, location, childcare, and cost. Similarly, participants reported their preference for less repetitive responses, more conversational continuity, and shorter messages. These recommendations mirror prior conversational-agent findings in the user experience—users can experience bonding and gratitude toward chatbots but may also feel frustrated when responses are repetitive and generic or when the agent misunderstands their input [63].

Additionally, our results support the findings of Liu et al [30], showing that GPT-powered chatbot-based PPIs can achieve high satisfaction ratings among participants. This supports the potential effectiveness of this architecture for specific clinical populations, such as those of caregivers of children with autism. Evidence from a free-text cognitive behavioral therapy conversational agent further suggests that conversational AI can sidestep stigma-related barriers while remaining engaging [63]. Finally, while Almulla and Khasawneh [27] validated the accuracy of LLMs for answering informational questions about autism, our study takes the next step by demonstrating that these models can also effectively deliver structured therapeutic exercises, not just information. All these findings complement web-based parent programs for ASD and other digital PPIs that have demonstrated improvements in caregiver well-being and self-efficacy. These results suggest that a brief strength-focused LLM chatbot can achieve user experience outcomes in line with broader digital mental health tools.

We also observed gains in WHO-5 scores. This finding mirrors that of previous studies showing that PPI can promote emotional resilience in stressed individuals [22,23], supporting the utility of a strength-focused approach for caregivers of individuals with ASD.

The lack of significant changes in depressive symptoms, perceived stress levels, and quality of life is likely due to the following two study characteristics: (1) the brief two-week dosage and (2) the small sample size (N=19). These factors possibly limited the statistical power and increased the risk of type II error. Meta-analytic work suggests a dose-response pattern, with longer PPIs yielding larger effects. Bolier et al [64] recommend at least 4 weeks and preferably more than 8 weeks; Sin and Lyubomirsky [65] likewise report greater gains with relatively longer durations. A more intensive or extended program with a larger cohort may therefore be required to influence deeper-seated distress and overall quality of life.

### Implications for Future Design

The interview findings translate into 4 refinement priorities. First, in terms of conversational naturalness and personalization, colloquial Cantonese should be used when appropriate, repetition and length should be reduced, and continuity should be improved by remembering prior inputs with consent. Second, in terms of flexible engagement and pacing, users should be allowed to choose sequences of exercises, break content into smaller chunks, and add reminders and a simple progress view to support adherence. Third, in terms of multimodal, interactive coaching, images and short videos (eg, illustrating savoring or strengths-in-action), and light, optional audio should be incorporated in practice. Fourth, in terms of ASD-specific guidance and navigation, brief, practical parenting techniques and links to community and professional resources should be provided. These directions are consistent with evidence that personalization and interactivity enhance engagement with digital health tools [66,67].

Accordingly, the next iteration (“Allie 2.0”) will transition to a multimodal LLM to handle text, images, and short video; expand the curated knowledge base with ASD-specific content; implement user-level memory and reminders with explicit consent; and strengthen guardrails.

### Limitations

This study has several limitations. First, the single-arm, nonrandomized design limits the ability to draw causal conclusions about the effectiveness of the intervention. We therefore interpreted changes in clinical outcomes as exploratory signals only and focused our primary inferences on feasibility and acceptability. Second, recruitment and data collection were challenging in this population despite our efforts in outreach through multiple channels. We ultimately enrolled a small sample of 19 participants. The high number of nonresponses and scheduling issues among eligible parents may lead to self-selection, meaning those who were more available or more comfortable with digital tools participated. This may have inflated our estimates of feasibility or acceptability. Third, the sample was composed entirely of mothers. This restricts the generalizability of the findings to fathers or other caregivers. Fourth, the intervention period was set at 2 weeks. However, while this reduced participant burden, this duration may have limited our ability to detect significant changes in depressive symptoms and quality of life. Fifth, we did not collect or analyze adverse events; no harm or disturbances were reported by participants. Finally, there was no long-term follow-up assessment in this study. These factors constrain the understanding of the sustained impact of the intervention and user adherence over time.

Future research should adopt RCTs to closely assess efficacy and comparative effectiveness. It is essential to expand recruitment across multiple sites, such as clinics, schools, and ASD service providers. Longer recruitment periods, flexible onboarding start dates, and reminders should also be considered to improve enrollment and relieve caregiver burdens. Additionally, it would be beneficial to include different caregiver groups, such as fathers and those from different cultural backgrounds. Extended follow-up periods would also provide deeper insights into sustained psychological impacts and adherence.

### Conclusions

In summary, this pilot study revealed that Allie is a novel, practical, and acceptable digital psychological tool for parents of autistic children. While these findings are preliminary, they point to the promise of chatbot-based interventions for improving caregiver well-being. They also highlight important areas for further technological improvement and thorough evaluation.

## Data Availability

The datasets generated or analyzed during this study are not publicly available due to data sensitivity; however, they can be obtained from the corresponding author upon reasonable request.

## Appendix

Multimedia Appendix 1 Intervention content based on the PERMA model (positive emotions, engagement, relationships, meaning, and accomplishment).

Multimedia Appendix 2 Example parent-Allie dialogue in figure 2.

Multimedia Appendix 3 Screenshots of participant’s flow of interaction with Allie.

Multimedia Appendix 4 Seven acceptability scores among the participants.

Multimedia Appendix 5 Paired dot plot of WHO-5 (World Health Organization–Five Well-Being Index) scores.

Multimedia Appendix 6 Data normality test results.

Multimedia Appendix 7 Characteristics of the 14 participants in the qualitative interview.

Multimedia Appendix 8 Most impactful exercises voted by participants.

Multimedia Appendix 9 Verbatim quotes illustrating helpful aspects and recommendations.

## Abbreviations

AES-256: Advanced Encryption Standard
AI: artificial intelligence
ASD: autism spectrum disorder
CONSORT-AI: Consolidated Standards of Reporting Trials–Artificial Intelligence
LLM: large language model
PERMA: Positive Emotions, Engagement, Relationships, Meaning, Accomplishment
PHQ-9: Patient Health Questionnaire-9
PPI: positive psychology intervention
PSS-10: Perceived Stress Scale-10
RCT: randomized controlled trial
SF-12v2: Short Form-12 Health Survey version 2
TLS 1.3: Transport Layer Security
WHO-5: World Health Organization–Five Well-Being Index
