# Supplementary material for: GPT-Powered Chatbot-Based Positive Psychology Intervention for Well-Being Among Parents of Children With Autism Spectrum Disorder: Single-Arm Mixed Methods Study
Source: JMIR Form Res. 2026 Mar 9;10:e85060. doi: 10.2196/85060 (PMC13010079; doi:10.2196/85060)
Supplement: Multimedia Appendix 1 [file formative_v10i1e85060_app1.docx]

Details of the eight core exercises in PERMA

| Exercise name | Exercise description |
| --- | --- |
| (i) Positive self-introduction | Participants were to compose a one-page introduction emphasizing their positive qualities. |
| (ii) Personal strengths | Participants were directed to assess their character strengths using the Character Strength Inventory and formulate realistic goals for applying these strengths. |
| (iii) Using personal strengths | Participants were instructed to design and execute activities that utilized their identified strengths. |
| (iv) Three good things | Participants undertook a week-long exercise, documenting three positive events each day. |
| (v) Gratitude letter | Participants were invited to craft a letter expressing gratitude or perform a gratitude visit. |
| (vi) Hope and optimism | Participants were guided to extract positive perspectives from challenging experiences. |
| (vii) Active or constructive responding | Participants were trained to respond to another person’s good news with clear positivity and enthusiasm. |
| (viii) Savoring | Participants were prompted to reflect on a positive moment, engage in mindful awareness, and share this recollection with someone close to them. |
