# Supplementary material for: GPT-Powered Chatbot-Based Positive Psychology Intervention for Well-Being Among Parents of Children With Autism Spectrum Disorder: Single-Arm Mixed Methods Study
Source: JMIR Form Res. 2026 Mar 9;10:e85060. doi: 10.2196/85060 (PMC13010079; doi:10.2196/85060)
Supplement: Multimedia Appendix 2 [file formative_v10i1e85060_app2.docx]

| Original dialogue | Translated dialogue |
| --- | --- |
| Parent:  「他是deadline fighter,只在考試前一週温習，中文是他掌握較好的科目，同學都考得相對地差，所以排名上有意外驚喜」 | Parent:  “He is a bit of a deadline fighter. He only studied during the week before the exam. Chinese is the subject he is relatively better at, and many of his classmates did not perform as well this time, so his class ranking turned out to be a pleasant surprise.” |
| Allie:  「聽起來,孩子在考試中取得了不錯的成绩，這讓你感到驚喜和欣慰。能夠在短時間内集中精力温習,並在考試中取得好成绩，這是一種很不錯的能力。這次的成功可能也讓孩子對自己的能力更有信心。  當你得知這個好消息時,你的心情如何呢?這件事對你和孩子之間的互動有什麽影響嗎?同時,這次的經驗是否讓你對孩子的學習方式有新的看法或啟發呢?  這様的經歷不僅能讓我們看到孩子的潛力，也提醒我們在生活中要多開關注那些讓我們感到驕傲和快樂的時刻。這是第一件好事，我們可以再来回憶一下還有没有其他讓你感到開心的事情呢?」 | Allie:  “It sounds like your child achieved a very good result in the exam, which made you feel surprised and pleased. Being able to focus, revise within a short period of time, and still do well is a very valuable ability. This success might also increase your confidence in his capabilities.  When you first heard this good news, how did you feel? How did this experience affect the way you and your child interacted with each other? At the same time, did it help you feel more assured that even though he sometimes gets easily distracted, he can still perform well and make progress?  Experiences like this not only show your child’s potential, they also remind us to notice those moments in life that make us feel proud and happy. This is already one ‘good thing’. When you think back over the past few days, are there any other things that made you feel happy?” |
| Parent:  「聽到這個消息時,其實我有些驚訝,因為無考慮過其他小朋友的分數是那様的不理想，相對之下他邊有比較出色的成績。我叫他不要驕傲，因為有時都有一些運氣的情况。」  另一些較為開心的事，是最近可以輕鬆地看電視制去舒緩一下 | Allie:  “When I heard the news, I actually felt a bit guilty, because I did not think about how other children might also have worked hard but did not get such good results. By comparison, his performance this time was quite outstanding. I told him not to become arrogant, because sometimes there is also an element of luck.  Another thing that has made me happy is that recently I have been able to lie down and watch TV dramas to relax a little.” |
