# Supplementary material for: GPT-Powered Chatbot-Based Positive Psychology Intervention for Well-Being Among Parents of Children With Autism Spectrum Disorder: Single-Arm Mixed Methods Study
Source: JMIR Form Res. 2026 Mar 9;10:e85060. doi: 10.2196/85060 (PMC13010079; doi:10.2196/85060)
Supplement: Multimedia Appendix 3 [file formative_v10i1e85060_app3.docx]

**Figure S1.** Landing page^a^.


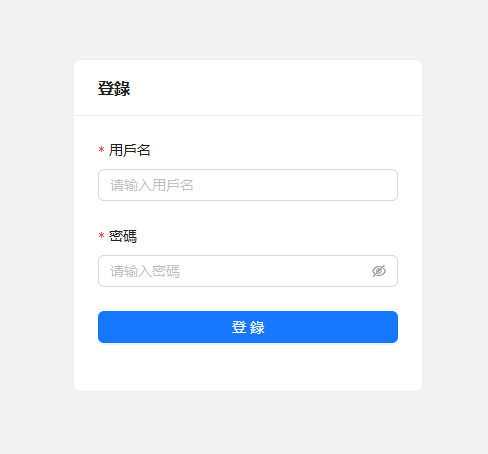


^a^Participant logs on using their user ID and password accordingly.

**Figure S2.** Home dashboard^a,b^.

^
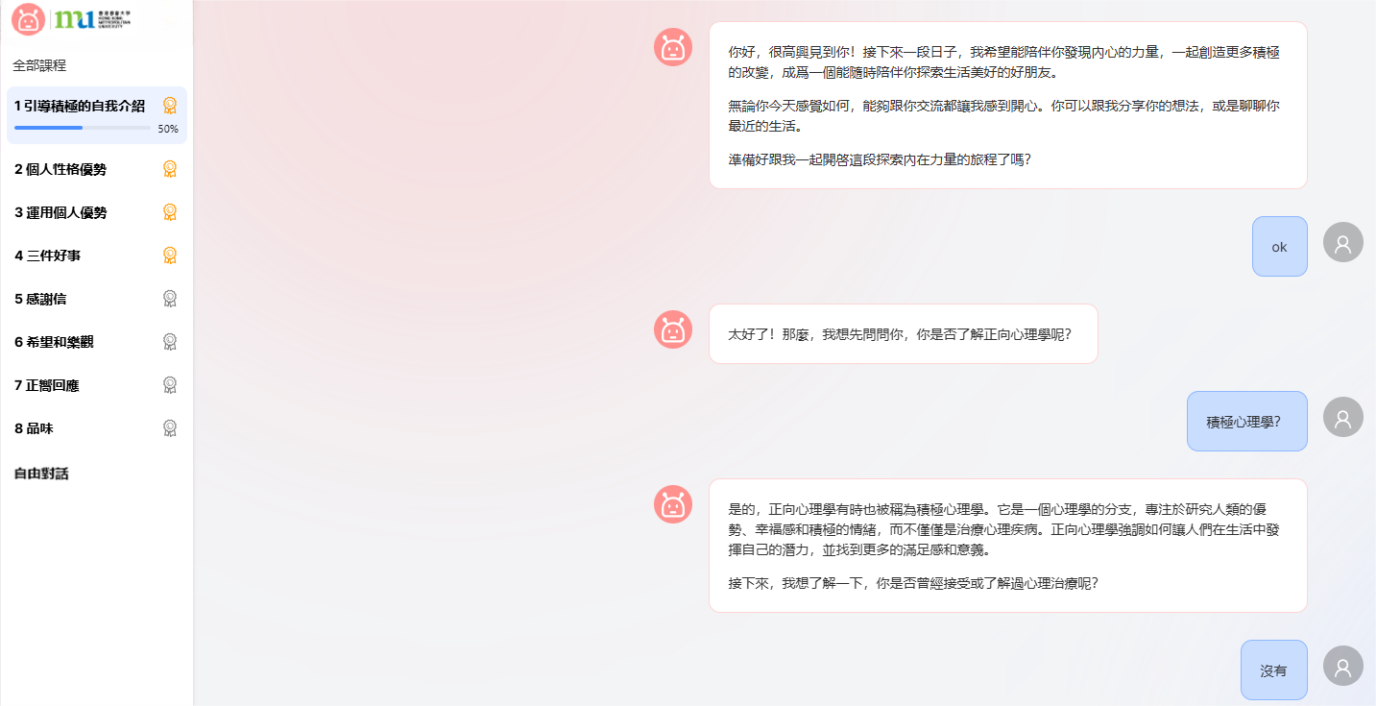
a^After logging in, participants can see each exercise on the left sidebar. They need to complete an exercise to unlock the next one. A progress bar under each exercise shows their progress. By reaching 100%, the badge in each exercise shall be lighted up.

^b^In exercise 1 (Introduction), Allie greets the participant, introduces the program’s focus on inner strengths and well-being. It also further checks the participant’s familiarity with positive psychology, then briefly explains it, and confirms the participant has no prior exposure.

**Figure S3.** Example interaction^a^.


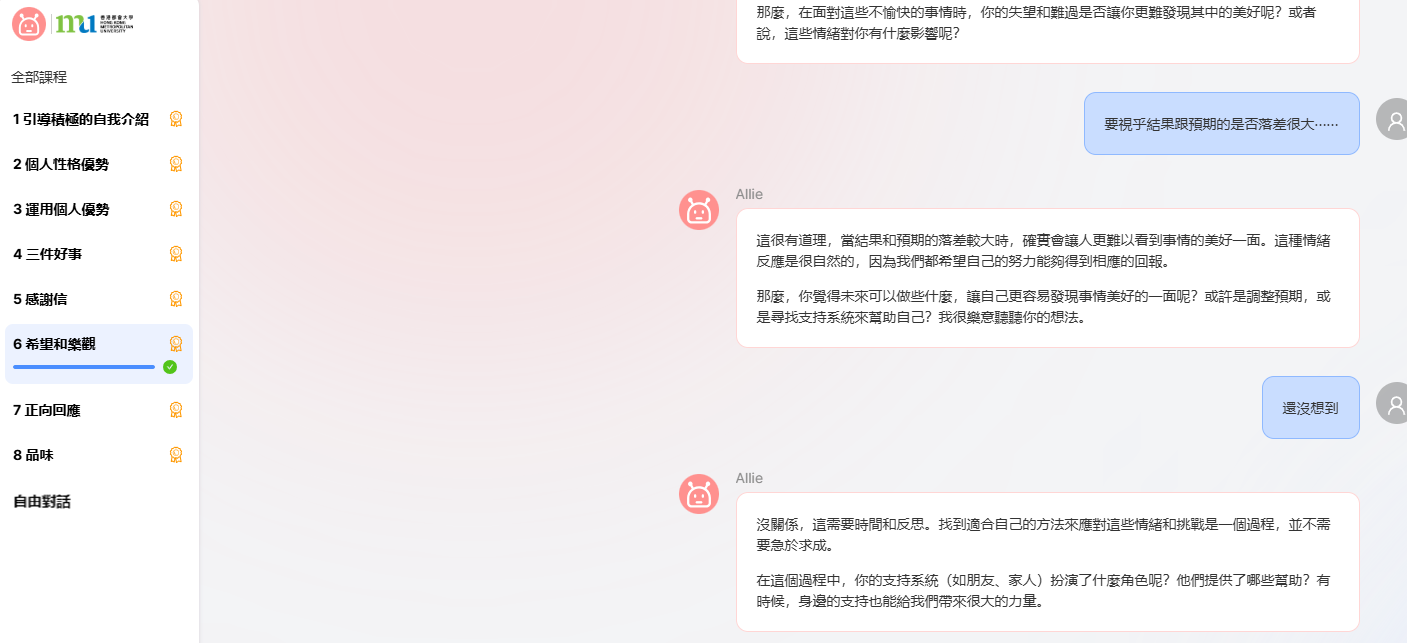


^a^Another example dialogue for exercise 6 (Hope and optimism), Allie validates the participant’s reaction to unmet expectations and prompts reflection on coping options like adjusting expectations and drawing on available social support.

**Figure S4.** End-of-exercise completion screen^a,b^.


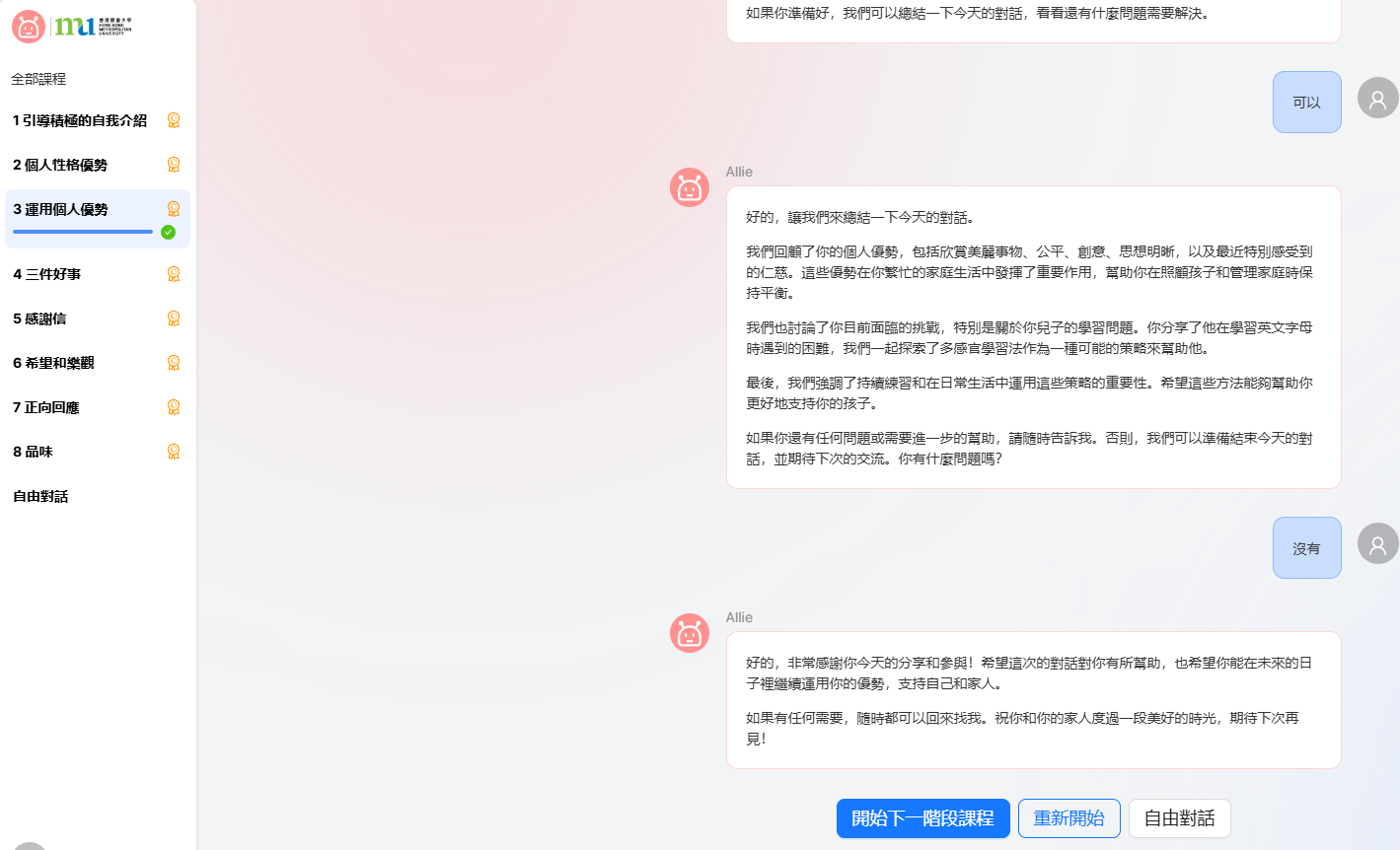


^a^Upon the completion of each exercise, Allie provides a brief recap of key points discussed and invites any final questions (example shown: exercise 3, Using personal strengths).

^b^After the recap, participants can select one of three options via buttons at the bottom: proceed to the next exercise, restart the current exercise, or enter free-form conversation.
