# Supplementary material for: GPT-Powered Chatbot-Based Positive Psychology Intervention for Well-Being Among Parents of Children With Autism Spectrum Disorder: Single-Arm Mixed Methods Study
Source: JMIR Form Res. 2026 Mar 9;10:e85060. doi: 10.2196/85060 (PMC13010079; doi:10.2196/85060)
Supplement: Multimedia Appendix 4 [file formative_v10i1e85060_app4.docx]

| Acceptability dimension | mean (SD) | % |
| --- | --- | --- |
| Overall satisfaction (1-7 scale) | 5.68 (0.7) |  |
| **Appropriateness of time** (1-7 scale) |  |  |
| Exercise duration (reasonable %) |  | 94.7 |
| Response time | 6.37 (0.68) |  |
| **Adaptability and availability** (1-7 scale) |  |  |
| Adaptability | 5.53 (1.07) |  |
| Availability | 5.79 (1.32) |  |
| **Information quality and trustworthiness** (1-7 scale) |  |  |
| Relevance | 5.82 (0.80) |  |
| Completeness | 5.53 (0.81) |  |
| Assurance | 5.80 (0.7) |  |
| **Affective and interpersonal perceptions** |  |  |
| Pleasure (1 - 5 scale) | 4.04 (0.77) |  |
| Empathy (1 - 7 scale) | 5.32 (1.15) |  |
| **Privacy concerns and usage intention** (1-7 scale) |  |  |
| Privacy concerns | 4.43 (1.43) |  |
| Usage intention | 5.68 (0.95) |  |

^a^Higher scores indicate more positive perceptions except for privacy concerns, where lower scores are preferable (n=19).
