# Supplementary material for: GPT-Powered Chatbot-Based Positive Psychology Intervention for Well-Being Among Parents of Children With Autism Spectrum Disorder: Single-Arm Mixed Methods Study
Source: JMIR Form Res. 2026 Mar 9;10:e85060. doi: 10.2196/85060 (PMC13010079; doi:10.2196/85060)
Supplement: Multimedia Appendix 6 [file formative_v10i1e85060_app6.docx]

| Measure | Time point | Shapiro-Wilk W | *P* value | Distribution |
| --- | --- | --- | --- | --- |
| PHQ-9 | Baseline | 0.901 | .05 | Marginally normal |
| PHQ-9 | Post-chatbot (end of intervention) | 0.893 | .04^a^ | Non-normal |
| PSS-10 | Baseline | 0.958 | .53 | Normal |
| PSS-10 | Post-chatbot (end of intervention) | 0.952 | .42 | Normal |
| WHO-5 | Baseline | 0.926 | .15 | Normal |
| WHO-5 | Post-chatbot (end of intervention) | 0.960 | .58 | Normal |
| SF-PCS | Baseline | 0.956 | .50 | Normal |
| SF-PCS | Post-intervention follow-up | 0.982 | .97 | Normal |
| SF-MCS | Baseline | 0.923 | .13 | Normal |
| SF-MCS | Post-intervention follow-up | 0.951 | .42 | Normal |

^a^*P*<.05 indicates significant deviation from normality.
