# Supplementary material for: GPT-Powered Chatbot-Based Positive Psychology Intervention for Well-Being Among Parents of Children With Autism Spectrum Disorder: Single-Arm Mixed Methods Study
Source: JMIR Form Res. 2026 Mar 9;10:e85060. doi: 10.2196/85060 (PMC13010079; doi:10.2196/85060)
Supplement: Multimedia Appendix 7 [file formative_v10i1e85060_app7.docx]

| Participant ID | Age | Gender | Education level | Employment status | AI experience^a^ |
| --- | --- | --- | --- | --- | --- |
| 1 | 42 | Female | High school | Self-employed | Never |
| 2 | 43 | Female | Bachelor's degree | Homemaker / unpaid family worker | Never |
| 3 | 40 | Female | Diploma or associate degree | Employee | Never |
| 4 | 46 | Female | Diploma or associate degree | Homemaker / unpaid family worker | Never |
| 5 | 44 | Female | Bachelor's degree | Homemaker / unpaid family worker | Never |
| 6 | 35 | Female | Diploma or associate degree | Homemaker / unpaid family worker | Tried only a few times |
| 7 | 44 | Female | Bachelor's degree | Employee | Never |
| 8 | 32 | Female | Diploma or associate degree | Homemaker / unpaid family worker | Never |
| 9 | 41 | Female | Bachelor's degree | Homemaker / unpaid family worker | Occasional use |
| 10 | 42 | Female | Diploma or associate degree | Employee | Never |
| 11 | 47 | Female | High school | Homemaker / unpaid family worker | Occasional use |
| 12 | 41 | Female | Bachelor's degree | Homemaker / unpaid family worker | Never |
| 13 | 51 | Female | Bachelor's degree | Employee | Never |
| 14 | 42 | Female | Diploma or associate degree | Homemaker / unpaid family worker | Never |

^a^AI = artificial intelligence.
