# Supplementary material for: GPT-Powered Chatbot-Based Positive Psychology Intervention for Well-Being Among Parents of Children With Autism Spectrum Disorder: Single-Arm Mixed Methods Study
Source: JMIR Form Res. 2026 Mar 9;10:e85060. doi: 10.2196/85060 (PMC13010079; doi:10.2196/85060)
Supplement: Multimedia Appendix 8 [file formative_v10i1e85060_app8.docx]

| Exercise name | First choice | Second choice | Third choice | Total |
| --- | --- | --- | --- | --- |
| Positive self-introduction | 4 | 1 | 0 | 5 |
| Personal strengths | 6 | 2 | 0 | 8 |
| Using personal strengths | 0 | 4 | 5 | 9 |
| Three good things | 1 | 2 | 2 | 5 |
| Gratitude letter | 1 | 2 | 0 | 3 |
| Hope and optimism | 1 | 2 | 1 | 4 |
| Active or constructive responding | 1 | 0 | 3 | 4 |
| Savoring | 0 | 0 | 0 | 0 |
| Total | 14 | 13 | 11 | 38 |
