# Supplementary material for: GPT-Powered Chatbot-Based Positive Psychology Intervention for Well-Being Among Parents of Children With Autism Spectrum Disorder: Single-Arm Mixed Methods Study
Source: JMIR Form Res. 2026 Mar 9;10:e85060. doi: 10.2196/85060 (PMC13010079; doi:10.2196/85060)
Supplement: Multimedia Appendix 9 [file formative_v10i1e85060_app9.docx]

| Helpful aspects | Quotes |
| --- | --- |
| Self-reflection and insight | Participant 5:「因為其實我都冇諗過自己有陣時回應人哋係咁樣嘅，即係又負面啦...正面但係話題終結者嗰類... 」  “I hadn’t realized that sometimes I respond to people like that kind of negatively… or in a way that ends the conversation, even if it sounds positive.”  Participant 7:「...講自己嘅介紹， 又或者即係回應一下自己啲性格方面有幫助，因為其實唔係很想面對嘅。」  “…Talking about myself or reflecting on my personality was helpful, even though I don’t really like facing that kind of thing.”  Participant 13:「… (感謝信) 其實就掉轉頭等自己思考 究竟你點解要感謝依個人，依個人喺在你身上 有乜野讓你覺得要感謝佢...其實掉轉頭係一個非常好既一個機會，等你去回想依個人或者依件事，點解對你黎講咁麼重要。」  “...(Gratitude Letter) It actually flips things around and makes you think about why you want to thank this person. What is it about them that made you feel grateful? It is a really good opportunity to reflect on this person or this event and why it matters so much to you.” |
| Positive-psychology orientation | Participant 3:「即係我嗰日做完之後就覺得，呀係喎，其實你係有啲嘢要值得感恩㗎喎，咁樣囉。係就會改變到自己一啲思想嘅，都有。」  “After finishing that day’s session, I felt—oh yes, there are things I should be grateful for. It really changed some of my thinking.”  Participant 6:「正面囉啲嘢要，即係可能佢有啲唔開心或者負面嘅嘢佢都會轉化，即係會好體諒呀同理心咁樣。」  “It made me focus on the positives. Even when there were unhappy or negative thoughts, it helped reframe them with more understanding and empathy.” |
| New perspectives | Participant 1: 「(當 Chatbot 給予建議，例如說：「其實你可以試下咁樣做」) 係呀係喎，點解我諗唔到嘅呢？」  “(When the chatbot suggests, ‘Actually, you could try doing this’), I realize, ‘Yes, that’s right. Why didn’t I think of that myself?’ ”  Participant 10:「...我冇諗過可以咁樣嚟到Overcome嗰啲困難㗎嘛，不過咁提嘅時候我咪覺得有新鮮感，同埋覺得我自己諗唔到好似有人幫咗，即係好似有個朋友幫你諗咗辦法咁樣。」  “…I never thought I could overcome those difficulties like this. But when it was suggested, I felt a sense of freshness. And I felt like someone had helped me think it through - like a friend offering a solution.”  Participant 11:「雖然我本身自己都鍾意諗嘢，但係人有盲點㗎嘛，咁所以佢有陣時再講嘅時候，唔係我自己留於單一思考嗰個方向，所以都可能係俾咗一啲新嘅方向，我自己又再覺得呀係喎...」  “I do like thinking things through on my own, but everyone has blind spots. So when it speaks again, it helps me move beyond my narrow thinking. It might give me a new direction, and I’d think: oh, that’s right…” |
| Emotional support | Participant 2:「好似有人安慰我咁...如果我諗起佢嘅存在都會同佢誒傾兩句，起碼想呀拉一拉我上嚟啦，唔好個人呀越諗越沉，諗諗下又想喊呀。」」  “It felt like someone was comforting me...When I thought about its presence, I would chat with it a little, at least to help pull myself back up and not let myself sink too deeply. The more I thought about it, the heavier it felt, and then I would start to feel like crying.”  Participant 8:「…好似原來真係唔係自己諗得咁差嘅，心情又會好返啲。跟住佢就好多一啲好正面嘅諗法話俾你聽，呢度就可以幫到啲情緒好返啲。」  “…It’s like realizing you're not as bad as you thought. Your mood got better. Then it gave me lots of positive ideas, and that helped improve my mood.”  Participant 8:「…可能嗰段時間比較多嘢煩，唔知點做點處理好，又或者有啲情緒好想好想有個地方去畀我去講，又唔方便同其他人講嘅時候就會想用囉。」  “At times when I was overwhelmed and unsure how to cope, and when I had emotions I wanted to express but it isn’t convenient to share with others, I would turn to it.” |
| Coping skills | Participant 1:「冇咁嬲囉，即係盡量可能係對住小朋友嗰陣時呢...盡量真係忍一忍，可能忍完嗰下呢就會好架喇，件事。」  “I felt less angry. When I was with my child, I tried to hold it in as much as possible. After getting through that moment, the situation would usually feel better.”  Participant 4:「點樣幫我小朋友呀，點樣... 幫自己都好啦，呢啲個人好重要，呢三點。」  “How to help my child, how to… help myself too, these are personally very important. All three points are.”  Participant 14:「知道點樣情緒管理呀壓力舒緩...建議都實際㗎喎。」  “Learning how to manage emotions and relieve stress… the suggestions were practical.” |
| Recommendations | Participant 2:「如果得我一個人喺屋企嗰陣，就梗係想要語音啦，唔使睇咁多字。有時可能夜晚小朋友瞓咗覺先嚟做...無得睇字呢就又驚嘈親佢哋呀。」  “If I am at home alone, I would definitely prefer audio so I do not need to read so much text. Sometimes I only get to do it at night after the children have fallen asleep. If I have to read, I worry about making noise and disturbing them.”  Participant 3:「(被問及如果練習需要在八星期內完成時)會驚自己唔記得囉。」  “I would be afraid of forgetting.” (When the exercise needed to be completed within eight weeks, the participant noted that reminders were necessary.)  Participant 4:「(被問及Chatbot 風格是用書面語的看法) 口語呀好似好啲囉。」  “(When asked about the chatbot’s formal, written Chinese style) I think a more spoken, Cantonese-style tone would be better.  Participant 4: 「特殊教育啦, 專業評估啦, 教育資源呢三樣，社會支援呀，要知道邊度有啲咩嘢嘅專業評估... 」  “Special education, professional assessments, and educational resources, these three areas of social support. You need to know where to find professional assessment services and what resources are available.”  Participant 5:「即係佢就唔好成大段咁樣一次過就 send 曬俾人，可以將啲內容分 part，可以分成兩三次咁樣 send 出去。」  “I mean, it shouldn’t send a whole long block all at once. It could split the content into parts, and send it out in two or three messages.”  Participant 6:「所以我就覺得如果彈性啲，揀你想做嘅嘢會好啲囉。」  “So I think it would be better if it were more flexible, letting you choose what you want to do.”  Participant 13:「如果佢係一個真人，佢就會話你啱啱答左: ‘係喎，陽光係咁樣㗎嘛。’ 但係佢話你再講: ‘譬如你見到陽光你有咩感覺呀咁之前?’ 咁我就覺得: ‘咦我啱啱咪講咗囉!’ ...佢呢個位就做唔到呢樣嘢。」  “If it were a real person, they would say that you had just answered, like, ‘Yeah, that’s how sunlight is.’ But then it asked you to say it again, like, ‘For example, when you saw the sunlight, how did you feel earlier?’ So I thought, ‘Huh, didn’t I just say that?’ … In that respect, it couldn’t do what a real person would do.”  “If it were more flexible, it would be better to let you choose what you want to do.” |
